# Five ways ‘health scholars’ are complicit in upholding health inequities, and how to stop

**DOI:** 10.1186/s12939-022-01763-9

**Published:** 2023-01-19

**Authors:** Sana Z. Shahram

**Affiliations:** 1 School of Nursing, Faculty of Health & Social Development, The University of British Columbia, Okanagan Campus Syilx Okanagan Nation Territory 1147 Research Road, V1V 1V7, P. 250 807 8685 Kelowna, BC Canada; 2 Canadian Institute for Substance Use Research, University of Victoria, Victoria, Canada

**Keywords:** Health equity, Complex systems change, Anti-racism, Anti-colonialism

## Abstract

Health scholars have been enthusiastic in critique of health inequities, but comparatively silent on the ways in which our own institutions, and our actions within them, recreate and retrench systems of oppression. The behaviour of health scholars within academic institutions have far reaching influences on the health-related workforce, the nature of evidence, and the policy solutions within our collective imaginations. Progress on health equity requires moving beyond platitudes like ‘equity, diversity and inclusion’ statements and trainings towards actually *being* and *doing* differently within our day-to-day practices. Applying complex systems change theory to identify, examine and shift mental models, or habits of thought (and action), that are keeping us stuck in our efforts to advance health equity is a promising approach. This paper introduces five common mental models that are preventing meaningful equity-oriented systems transformation within academia and offers ideas for shifting them towards progressively more productive, and authentic, actions by health scholars to advance health equity across systems.

## Introduction

Advancing health equity is a ‘wicked problem’; equity issues are interactive, complexly inter-related, context-dependent and dynamic, without clear-cut endpoints or obvious solutions [1]. Equity work is messy and complex work- fundamentally at odds with much of health scholarship’s reliance on bounded, biomedical, linear and positivist approaches to problem solving [2]. This clash mirrors cognitive dissonance between the enthusiasm with which health scholars^1^ critique health inequities and our comparative silence on the ways in which our own institutions (and our work *within them*) recreate and re-entrench the power distributions and dynamics and systems of oppression at the root of those inequities. The behaviour of health scholars within academic institutions have far reaching impacts; we shape how we make sense of health, how health systems and professionals behave within them, and importantly, what is and is not paid attention to within the domain of health equity ‘work’.

Despite a welcome shift in mainstream attention since 2020, spurred in part by George Floyd’s public murder, progress on health equity remains slow within our institutions. For health scholars, health equity is mostly still a problem that is ‘out there’ but never ‘here’ [3]. Our own institutions, identities and roles remain largely absent, unnamed and unacknowledged in most of our work, and demanding foundational changes of our institutions and ourselves somehow remains beyond our imagination of scholarly activities. As an early career, cis-hetero-female tenure-tracked Faculty member, who is a non-Black, non-Indigenous racialized child of Iranian and Muslim immigrants, my identity belies the complexity of many peoples’ positionality; most of us find ourselves simultaneously benefitting from and suffering from various structures of oppression [8]. My experiences of racism, for example, are also embedded within the benefits of my relative proximity to whiteness and the ways in which I have been positioned historically to be complicit in anti-Black and anti-Indigenous racism. Recognizing these complexities, and accepting the limitations inherent in a journal article, I have chosen to direct this article at our shared and collective identities as ‘health scholars’. I offer that ‘we’, by virtue of occupying the privileges afforded to us through our positionality as health scholars, also have a shared set of responsibilities and accountabilities *within* our own institutions, as a minimum starting point for sustained action on health inequities. Although our personal positionality and relative power within these institutions is vitally important to how we might act on these responsibilities individually, this discussion is beyond the scope of this paper, offered here as an initial invitation to deeper, systems-level thinking about our collective roles in maintaining health inequities.

To date, systems-blind and superficial efforts have dominated our institutional attention, while “detailed and often mundane work of noticing what is invisible to many” at the heart of real progress has been largely absent [4]. The popular systems-change parable of a fish swimming up to another fish to ask ‘how’s the water’? only to have the other fist stare back blankly and respond, ‘what’s water?’ is an apt summation most equity, diversity and inclusion efforts. And yet, we are all swimming in the proverbial ‘water’ of systems, and our inability (or unwillingness) to see, and therefore shift, the currents of that water, keeps us perpetually stuck in advancing equity within health systems [4]. Systems thinking attunes our work to ensure it results in the design and implementation of solutions to the conditions that hold a problem in place. This is possible via more nuanced and context-sensitive strategies for addressing power imbalances and fostering the dynamics of collective action for political reform [3, 4]. There are three levels of systems change that exist to varying degrees of visibility to players in the systems (from explicit to semi-explicit to implicit, respectively): **(1)** structural changes, focused on shifting policies, practices and resource flows (explicit); **(2)** relational changes, focused on shifting relationships & connections and power dynamics (semi-explicit); and **(3)** transformative changes, focused on shifting ‘mental models’ (i.e. the deeply held beliefs, assumptions and taken-for-granted habits of thought that impact how we think, what we do and how we talk) (implicit) [4, 6]. These six conditions are mutually reinforcing or counteracting, and can reinforce inequity by upholding and perpetuating the structural and systemic roots of white supremacy [4].

Every level of systems change requires careful attention to the ways in which they can recreate inequities. Particularly when considering transformative change, it is essential to recognize that mental models: create implicit biases through which individuals interpret and make sense of other people, ideas, and events; dictate where we focus our gaze in terms of understanding a problem, and our imagined solutions to that problem; and, are often shaped by those who hold the most power [3, 4]. Mental models are therefore not happenstance, naturally occurring or benign; in the context of settler states, like what is now known as Canada, they allow the subjugation, extraction and exploitation of the global majority to continue unabated, while simultaneously obscuring our complicity as power-holders who embody and benefit from these systems. In terms of our responses within academic settings to calls for addressing equity, racism and justice, mental models that entrench a culture of ‘changelessness’ and perpetuate the status quo within our institutions and among ‘health scholars’ are similarly *explicit* (though often insidious) tools for advancing white supremacy. Transformative change in our institutions therefore demands new capacities for combining scholarly, operational and political work to name, disrupt and shift the mental models sustaining the complicity of health scholars in advancing health inequities [4–6]; in other words, we actually have to *be and do* differently, as individuals and as institutions of ‘higher learning’ to make meaningful progress towards health equity. This paper introduces five interrelated and reinforcing mental models keeping health scholars ‘stuck’ in our efforts to advance health equity across health systems alongside solutions to get us ‘unstuck’.

### Mental Model 1: Euphemisms

Euphemisms are mild or indirect words or expressions substituted for those considered too harsh or blunt when referring to something unpleasant [7]. It refers to the tendency to understate or downplay the root causes of inequities by obscuring the role of systems, institutions, and ourselves in perpetuating and advancing systems of oppression. Some common examples of euphemisms include substituting: equity, diversity and inclusion (EDI) for racism and anti-racism; vague ‘BIPOC (Black, Indigenous, and People of Colour) issues’ for white supremacy or people racialized by whiteness; or, ‘empowerment’ initiatives for ‘equity-seeking’ folks for the processes through which people are systematically and deliberately disenfranchised.

Tiptoeing around the causes of inequities through the use of euphemisms renders the design of meaningful solutions impossible. When perpetually focused on the outcomes of oppressive systems (i.e. ‘vulnerable’, ‘marginalized’, ‘equity-seeking/deserving’ people), our gaze will *never* land on the actual problem of systems that provide unearned advantage to some while creating unearned disadvantage for others [2, 8]. For example, many have ubiquitously adopted the language of EDI, with little regard to defining whom we see as ‘diverse’ and, in what, exactly, we are including them. Left unchallenged, our understanding of success in addressing issues of equity is limited to aspiring to ‘include more non-white people’ within (by our own implicit admissions) harmful, exclusionary, violent and racist spaces. EDI narratives falsely locate the problem as the absence of certain populations within our institutions, while abdicating us from addressing our participation in and collusion with systems that exclude them.

Euphemisms create self-fulfilling prophecies on the futility of efforts to advance equity. While we spend time, money, resources and efforts mobilizing committees, strategies, public statements and trainings targeted at palatable, gentler, and non-inflammatory goals, euphemisms mean that we ignore our flawed central values at the heart of the problem [2, 9]. When these efforts inevitably prove ineffective: momentum wanes and progress is deemed impossible; the list of efforts are used by leaders to make false claims of having ‘solved the equity issue’; and/or, those who speak up (usually those most impacted) are deemed unreasonable, ungrateful and unlikely to be satisfied.

Euphemisms belie our collective discomfort with conversations around equity, race and justice, while also amplifying it through conversations focused on the *wrong topics*. Demands for ‘more resources’, ‘workshops’ and ‘glossaries of equity terms’ presume we have a right to occupy a central role in the telling of stories and perspectives of groups to whom we do not belong [10]. Constant discomfort to engage in conversations not meant for us is unsurprising- our entitlement to study, name, describe and analyze others clouds our ability to see the shortcomings and arrogance of our efforts [10]. Interrupting euphemisms by instead naming and attending to issues related to the (mal)distribution of power within our own institutions, for example, allows us to identify and engage in the conversations that are *rightfully ours* to lead.

Shifting this mental model requires a commitment to becoming truth tellers who use specific and precise language to talk about: (1) the structures that create privilege; and, (2) the people who *benefit* from that structure [8]. By shifting our gaze to ourselves, our own structures and our systems, we can stop waiting to learn enough about some imagined ‘them’ and instead start building systems that no longer create unearned advantages for only a few. So, rather than the ‘underrepresentation of BIPOC faculty’, we discuss the massive *over*representation of white, heterosexual, cis-gendered, able-bodied men in leadership positions [9]. Engaging in critical white studies, to confront whiteness, and its impact on our collective well-being, through thorough and frequent assessments of our institutional practices, pedagogies and every-day interactions, becomes our task [9, 11]. Interrupting the use of euphemisms in our own spheres of influence (i.e. our own writing and teaching, meetings, etc.) through a commitment to specific and precise language refocuses our gaze on the most significant issues of “white people who continually act in the preservation of racism [rather] than an emphasis on diversity and inclusion and the lack of BIPOC within [our] institutions” [9]. Another way these acts of preservation manifest is through our preoccupation with the ‘middle ground’.

### Mental Model 2: Middle Ground

The ‘middle ground’ refers to a standpoint or area midway between extreme or opposing positions, options or objectives [12]. Like euphemisms, our collective commitment to avoiding conflict fuels the middle ground mental model, where remaining neutral often comes at the expense of justice. Some common examples of the middle ground include the seemingly endless calls for ‘hearing all sides’, ‘being reasonable’, ‘not getting political’ or ‘conducting thorough investigations’ in response to claims of racism or discrimination within academic institutions, with tangible action rare. Not to be confused with compromise, which is, by definition, reached through *mutual consent* of opposing parties [13], the middle ground is instead a more insidious feature of being trained within white supremacy culture that prioritizes being seen as good and polite above being seen as just (https://www.whitesupremacyculture.info/).The middle ground is a default frame that perpetually squashes progress while feigning benignity- it forgets that all the middle ground between oppression and justice is still oppression.

The middle ground mental model mirrors critique that has plagued health scholarship for years. Steeped in dominant biomedical, positivist, and largely atheoretical approaches, health scholarship often positions ‘knowledge’ as objective truth while failing to engage in the political project that is at the heart of health equity work [2, 14]. This manifests in health researchers’ tendency to quantify, describe and *mystify* inequities, without problematizing or intervening on the systems-level conditions that are driving the unequal distribution of resources and opportunities for good health [15, 16]. These practices rely on largely uncontested assumptions that the measurement and reporting of inequities is somehow objective, scientific and valid, while the lived and living experiences, as well as broader sociopolitical contexts, behind those data points are comparatively subjective, unreliable and outside the scope of our work as scientists. Every facet of health research systems perpetuate and recreate these fallacies, including the publishing practices of health journals that heavily skew the presentation of findings away from the necessary task of disrupting the root causes of health inequities [15]. When we are apolitical in our craft, it is rather unremarkable, then, that we are apolitical in our day-to-day.

Our aversion to conflict or ‘taking sides’ when it comes to equity issues keeps our work perpetually stuck by manufacturing the appearance of debates where they do not exist. When objectivity conflates with neutrality, we create, maintain and reinforce false equivalencies. For example, much time, energy and general ‘fussing’ is spent attending to the discomfort, needs and wants of primarily white faculty so that they may be able to ‘navigate’ discussions around race, equity and justice in ‘safe ways’. Administrators, leaders and colleagues falsely equate the personal discomfort of (often tenured and PhD-prepared) life-long learners in engaging in *professional development* with the struggles of people literally *fighting for their lives.* The ‘feelings’ of the privileged trump the impacts of our violence.

Rejecting the middle ground requires us to reject liberal incrementalism-reformist approaches to social justice and to engage instead in articulating more explicit policy agendas, based in evidence, and to apply them to our own day-to-day systems^2^[9,14]. Ironically, it demands that we embrace both pragmatism and abolition, to elevate our collective gaze towards systems, rather than people, to engage in constant evaluation of the conditions that oppress and marginalize people (C. Horsethief, personal communication, February 5, 2022). It requires us to be kind, instead of nice. As Luvvie Ajay Jones distinguishes in her 2020 book *Professional Troublemaker*, being nice is telling someone that it is raining outside, while being kind is handing someone an umbrella [17]. Kindness requires us to see an issue, *and* make a claim or judgment on the impact of that issue *and* react to interfere with or alleviate its impact. Kindness is fundamentally at odds with the middle ground’s passive assumption that equity work follows continual linear progress [9]. To paraphrase Martin Luther King Jr’s *The Other America*, social justice has never rolled in on the wheels of inevitability, but from the hard work of dedicated (often-racialized or otherwise oppressed) individuals [18]. Shifting away from the middle ground also requires us to interrupt distractions.

### Mental Model 3: Distractions

Distraction refers to something that prevents someone from giving their full attention to something else [19]. Distractions manifest as the activities, actions and preoccupations that keep us ‘busy’, take time, money, and resources, but do little to advance tangible equity action. Examples of distractions include undergoing extensive (and often redundant) strategic planning processes, or the constant pursuit of new data. In Toni Morrison’s keynote for Portland State in 1975, she shares her frustration with the functions of this mental model in distracting us from doing the work needed to advance our collective liberation:

*The function, the very serious function of racism is distraction. It keeps you from doing your work. It keeps you explaining, over and over again, your reason for being. […] None of this is necessary. There will always be one more thing.*

Distractions are rooted in an over-reliance on a narrative of individual benevolence (i.e. the construction of power holders as implicitly well-meaning people who have just not had access to the right information, in the right way yet, to make better decisions) as the catalyst for systems change [20], rather than the required and essential work of redistributing power and resources.

In 2020, following the broadcasted murder of George Floyd, resources and attention shifted to anti-racism work, but often only in ways that belied how little our institutions understood (or more likely, *valued*) equity, diversity and inclusion. The outcome was what I affectionately refer to as, *Health Scholarship*, *The Remix*. (We change the music, but the dance steps have largely stayed the same). Special issue research funding, for example, for projects related to equity, racism and social justice, has sky rocketed. While worthy, the topical shift often also confusingly doubles as a named institutional strategy for including more ‘diversity’ in research teams. Apparently, there are no black chemists, for example- we simply have an issue with funding the right topics to attract the singular interests of racialized scientists. While I am being overly pedantic, it is purposeful to reveal the insidious ways white supremacy permeates everything we do.

The cognitive dissonance at the heart of these distractions are behind many of the baffling inconsistencies in major equity strategies. For example, implementing mandatory sex and gender training modules for funding applications to the Canadian Institute for Health Research over a decade *before* offering gender inclusive submission forms for applicants. Or, assembling a fully white Board for the Canadian Strategies for Patient Oriented Research, created specifically to address the lack of diverse voices in health research. Or, special journal calls for topics related to racism, equity and justice without: structural changes to ensure more voices can participate and thrive in these top tier journals (on this *and* other topics shaping our landscape of understanding, evidence and knowledge); compensation for folks without tenure-track faculty positions to contribute; or, word length and style flexibilities to support other knowledge contributions. Without these practical (and often procedural) changes, rather than shifting systems, we are simply getting more creative in the ways we extract knowledge and lived experience for our collective gain and pageantry.

Distractions are fueled by white people’s needs, rather than the demands of racialized people, increasingly filling all of the space created to advance equity since 2020; meanwhile, the radical restructuring of the university, and excavating our institutional histories to identify tangible ways to make reparations and restitutions, have remained outside the realm of consideration [9, 20]. Trainings and workshops have deflected equity work into psychological self-help and human resources discourses while simple actions for decolonizing and social justice (i.e. eliminating tuition, forgiving student debt, reparations) have been masked, or rejected as too radical and unfeasible [9]. These distractions are further manifested through the oft-quoted and egregious misnomer of white folks being told ‘do the work’. Racialized scholars, activists and communities have *already done the work*, with grace that is undeserved, of offering reports, calls to action, testimonies, art, stories and direct and practical recommendations for pathways forward [21, 22]. Our demands for more evidence, content and information that centres our voices, using our dominant frames, to re-tell and re-story the experiences, pain and humiliation of others, has remained insatiable [20].

Our constant calls for better knowledge translation practices also keep us perpetually distracted. We double down on an unsubstantiated assumption, and unreliable theory of change within the academy, that more data will contribute to social justice solutions, as if good evidence alone drives political choices (and the distribution of power and resources) [9, 10, 14, 20]. These costly and time-consuming distractions continue to fetishize the pain and trauma of excluded communities, with no connection to action, while exposing the absurd lengths we will go to in order to escape accountability [20]. What remains less valued is unpacking our complicity in enacting that pain- the mental model of distractions allows us to remain comfortable external observers, always busy *doing the doing*, rather than as the rightful subject of inquiry- to understand why more academic knowing has not innately contributed to the advancement of equitable health outcomes [20].

The antidote to distractions is to refuse continuously moving towards shifting goal posts- saying no to the calls for a new report, survey or committee. To remain wary of superficial strategies offered by administrators that engage in organizational symbolic impression management, or social justice projects that rely on institutions of power to ‘do the right thing’ [9, 10, 20]. As racialized and excluded communities increasingly refuse to participate in routes to justice that require appealing for their own humanity, we are urgently being called upon to stop pandering to the trope of benevolent power holders to instead engage in the urgent work of redistributing power, resources and opportunities while making reparations and restitutions in response to the priorities of communities and Nations harmed by our institutions [10]. Doing this work requires lifting the veil of mystery that surrounds the operations of academic institutions.

### Mental Model 4: Mystery

Mystery describes something that is difficult or impossible to understand or explain [23]. In response to injustices within our institutions, mystery manifests through formal and informal statements that locate problems *nowhere.* Not only is the role of power in advancing equity often misunderstood within our research and teaching activities, it is tacitly absent and ignored in our day-to-day activities. We somehow simultaneously occupy the space of the ‘authority’ and ‘knowing body’, and yet expertly manage to evade any obligations to fix any problems. This paradox translates to bloated, risk averse, administrations that are skewed heavily towards deflection and inaction [20]. Responses of ‘it’s too complex’, ‘it’s out of my hands’ or ‘that’s above my paygrade’ define a culture that is implicitly satisfied to act in the preservation of racism and injustice, so long as it allows us, and the power and influence we hold, to remain abdicated, absent and unseen.

Uncontested and unsubstantiated assumptions of universities as moral by nature sustain this mental model [9, 10, 20]. Yet, academia’s foundational ties to oppression mar these claims; for example, universities generated the theories and justifications of racism and subjugation that have dispossessed Indigenous peoples, and we reconstruct these practices through present-day activities within our institutions [9, 24]. As health scholars, we actively contribute to (and benefit from) the de-legitimization of non-white knowledges, while setting white cultural standards for what is valid, rigorous and worthwhile, in both past and present [9]. Our tolerance of mystery, and lack of accountability, perpetuates uncontested white privilege [9].

We must move beyond fighting for supportive environments from our administrations to instead demanding a university that “not only promotes but also models social and economic justice” [25]. We need to see ourselves as active participators and as the *site for struggle and change*, to ‘dig where we are’, before (or at the very least alongside) our efforts to solve ‘other’ problems [26]. All of us, including our leadership, must protect our collective capacity to be *in*, but not *of*, the university [9, 20].

### Mental Model 5: Capacity

Capacity refers to the ability or power to do, experience or understand something [27]. Misgivings about our own capacity belie previous mental models’ convincing lure to spend most of our time and energy proving our own ‘goodness’; the important *but personal* work of attending to individual emotions of guilt, shame, anger and discomfort subsumes our collective work of dismantling white supremacy [28]. The capacity mental model feeds hysteria that causes even the most grounded among us to forget basic skills and training at the mention of race or racism (e.g. in 2020, UBC’s President, Santa Ono, committed all of us, trained scientists, to the lofty goal of somehow *eliminating* bias, rather than the more reasonable task of accounting for and addressing harmful bias in our systems) [29]. It is rooted in a false narrative that equity work lies perpetually beyond our collective capacities, both in skill and time. Nevertheless, we remain the most prepared, most protected and most compensated people in all of society to do this work. *If not us, then who?*

As health scholars, training future leaders, we perpetuate our values across health and healing systems, for better or worse. We do this overtly, through the content we teach, but also covertly through the every-day behaviours we tolerate and condone. Cultures that dictate overlooking bad behaviour by students and colleagues (like hostile communications or emails) in the pursuit of positive teaching scores, tenure reviews and ‘collegiality’ reveal that we have forgotten to whom our work belongs. In Canada (and many countries), our salaries, research funds and even training are primarily publicly funded. Accountability rubrics ought to reflect this; to reward serving society, rather than the neo-liberalized university and its agendas. Remaining silent in our own schools, classrooms and faculty meetings, while protected under tenure, implicates us as culpable in amassing reports of harmful, racist, flailing and unjust health systems [9]. Advancing the health of populations without engaging in activism within our own institutions is impossible.

Scholarly critique, devoid of organizing and action, can comfortably co-exist with the professionalized, market-based, capitalistic academy: “such scholarly publishing […] intrudes on the space for challenging its legitimacy [9]. ‘Raising awareness’ through extracting data from over-coded communities to accumulate theory allows us to maintain a sense of social exteriority that imagines knowledge production, with no meaningful application, as a form of change in itself [9, 20]. In Luongo’s (2021) auto ethnography of navigating (and being expelled by) critically-oriented social sciences scholarly spaces while being codified as ‘mentally ill’ or ‘addicted’, she expertly outlines how we ‘cut out’ the identities we claim to research and serve, while simultaneously building careers out of this exclusion [30]. Applying our very own theories in our own contexts somehow falls outside of our perceived capacity. Advancing equity urgently requires new collective capacities to undo the ways in which we contribute to the ‘ivory tower’ of our spaces, removed from the practicalities of the real world, and the rightful accountability to those whom our work truly belongs.

We need fundamental changes to the ways in which we understand our work. As detailed in Dr. Stephanie Nixon’s recent contract for a new Director role in Health Sciences, our leaders must be “mandated to advance the transformation of internal structures […to embed] equity, inclusion and anti-racism into education, research and care […] to make way for imagining and bringing to life a different future within and beyond the health sciences that is more just and peaceful for all” (Nixon, Personal Communications). We must trade self-promotion and recognition for our personal work to instead embrace collectivity, reciprocity and mutuality to connect to the communities we are involved in, and to each other [9]. We need to adjust all of our accountability rubrics and day-to-day actions towards serving the public. To make all of us, including our own institutions, better. Rather than DEI committees, might we imagine committees that shift our goals and actions to work for the interest of the public? Might we create terms of reference for how we commit to ongoing and fearless critique of our institutions rather than ahistorical equity efforts that reproduce and retrench narratives of helplessness and changelessness within our institutions [9]? Might we reimagine our identities to see the university as access to material and resources without understanding it as in and of itself a place for enlightenment? Might we stand in solidarity to reject university cultures that reward socially isolated faculty whose skepticism and claims of objectivity *leave the world as-it-is* [9, 10, 20, 24]? Beyond checklists, trainings, workshops and committees, equitable systems transformation relies on our willingness to lean into the social justice interventions at the heart of these superficial efforts. It relies on: collective organizing; redistributing power downwards; conducting white supremacy audits of our systems; conducting collective participatory action research on our own institutions; and, ultimately, committing to the ongoing work of becoming more truthful about our roles in advancing or interrupting inequities, both with ourselves and with others [9, 10, 20].

## Implications & Opportunities

The COVID-19 pandemic has exacerbated cracks within our collective systems, with health and public health systems at the forefront. Sustained and collective pandemic responses among segments of the population have been challenging, flamed by troubling trends of COVID-19 deniers, conspiracies, populism, and the emergence of the age of ‘disinformation’. While unpacking all these trends is beyond this paper’s scope, what remains pertinent is the cognitive dissonance it has exposed among health scholars, and our inability to adequately hold ourselves, and our systems, to account. While our work and our institutions implicitly and explicitly perpetuate and reinforce neoliberal ideas of individuality, market justice and meritocracies, we simultaneously *bemoan and chastise* people for having no frame of reference to process the collectivity pandemic responses require of us. Those who cannot or will not participate in alterations of their daily life in the name of collective preservation are the canaries in the coalmine, signaling the abject failure of our systems to prepare us meaningfully for the work of thriving societies. Any retreat from these truths is willful ignorance that is tantamount to that which bewilders many in the ‘COVID-deniers’.

The mental models offered here are not exhaustive and there are likely more to consider. As increased activism by students and faculty alike brings societal protests into the university context, we must adapt or perish [9]. Surface promises and actions further crack the veneer of the university project, and maintaining our relevance requires a humble commitment to change, both incrementally and structurally- despite possibly finding the project futile [20]. Confronting these mental models attunes us to developing future shared commitments in service of our goals through everyday, constant and ongoing practices that fortify health scholars towards our aspirational moral roles in society.

## Conclusion

Equity work at its core is radically hopeful. The concept of health equity is contingent upon a belief that inequity is human-caused and therefore human-solved. Belief in the possibility of health equity implicitly believes that health scholars must change by doing more than just thinking and talking about health equity. Interrogating and interrupting mental models that sustain inequity opens opportunities to engage in actions with emancipatory potential to align our practices with our aspirational values:

*There is no legitimacy to the field of education if it cannot meaningfully attend to social contexts, historical and contemporary structures of settler colonialism, white supremacy and anti-blackness. Social justice is not a catchall; it is the all* [20].

## Data Availability

N/A- comment.

## Footnotes

1 For the purposes of this paper, ‘health scholars’ is inclusive of anyone who plays a tacit role in the advancement and shaping of formalized health ‘knowledge’ within an academic setting (i.e. researchers, instructors, tenure-track and tenured health sciences professors, clinical faculty). Single quotations acknowledge that while we have all been named by our institutions as ‘health scholars’, our identities are embedded within problematic structures that do not hold singular authority for defining or anointing scholars, and the term itself warrants interrogation.

2 A note of caution against swinging the pendulum further towards nihilistic positions, and virtue signalling practices, of individually ostracizing those who reasonably apply complexity and nuance to contentious issues (C. Horsethief, personal communication, February 5, 2022).
